# Global crop waste burning – micro-biochar; how a small community development organization learned experientially to address a huge problem one tiny field at a time

**DOI:** 10.1186/s42055-020-00037-y

**Published:** 2020-11-23

**Authors:** Michael Shafer

**Affiliations:** 1 Warm Heart Foundation, 61 M.8 T.Maepang A.Phrao, Chiang Mai, 50190 Thailand; 2 Warm Heart Worldwide, Inc., 434 Cedar Avenue, Highland Park, 08904 NJ USA; 3 Political Science (Emeritus), Rutgers University, New Brunswick, 08901 NJ USA

## Abstract

The world’s 2.5 billion poorest people - small farmers living at the far fringe of the developing world – and their billion or so slightly better off neighbors burn 10.5 billion metric tonnes (tonnes) of crop waste annually. Smoke from their fires reddens the sun, closes airports, shuts schools and governments – and kills millions of people (World Health Organization (WHO). who.int/health-topics/air-pollution#tab=tab_1). Their fires release 16.6 billion tonnes of CO_2_, and emit 9.8 billion tonnes CO_2_e, 1.1 billion tonnes of smog precursors and 66 million tonnes of PM2.5. (Akagi et al., Atmospheric Chem Physics 4039-4071, 2011; Environmental Protection Agency, epa.gov/ghgemissions/understanding-global-warming-potentials; Food and Agriculture Organization, FAOSTAT, http://www.fao.org/faostat/en/#data) [See Attachments 1–3. For details of the Attachments, please see the section below entitled “Availability of data and materials.”]. No one yet has stopped the burning. Seminars, health warnings, bans, threats, jailings, shootings – nothing has worked, because not one has offered farmers a better alternative. This is the story of how Warm Heart, a small, community development NGO, learned enough about small farmers’ plight to collaborate with them to develop the technology, training and social organization to mobilize villages to form biochar social enterprises. These make it profitable for farmers to convert crop waste into biochar, reducing CO_2_e, smog precursor and PM2.5 emissions, improving health and generating new local income – in short, to address the big three SDGs (1, 2 and 3) from the bottom.

Warm Heart, however, wanted more; it wanted a system so appealing that it would spread by imitation and not require outside intervention. Based on what it has learned, Warm Heart wants to teach others that the knowledge to stop the smoke and improve the quality of one’s life does ***not*** require outside experts and lots of money. It wants to teach that ***anyone*** can learn to create a more sustainable world by themselves.

This article traces the experiential learning process by which Warm Heart and its partners achieved their goals and shares Warm Heart’s open source solution. It serves four purposes. The article closely explores an experiential learning process. It details the underlying logic, workings and consequences of crop waste burning in the developing world. It demonstrates the application of this knowledge to the development of a sustainable – even profitable – solution to this global problem that does not require costly outside intervention but can be undertaken by local communities and small NGOs anywhere. Finally, it models how local communities, small NGOs and social investors can turn this global problem into a profitable business opportunity.

Today the Warm Heart website’s hands-on oriented environment section averages 400,000 unique visitors annually; visitors are particularly interested in its deliberately accessible information and collection of short videos about how to make cheap biochar machines and biochar products [1]. Visitors come from the developing world – India, Pakistan, Iran, Africa. Warm Heart practices what it preaches. Where we live in the mountains of rural North Thailand, we train small farmers. We buy every kilogram (kg) of biochar that local farmers make; test, make and sell biochar-enhanced potting soil, compost, fertilizer^1^ and briquettes.^2^ We operate three biochar social enterprises as part of our ‘Stop the Smoke’ campaign, the only effort in Thailand to eliminate the choking smoke from annual crop waste burning [2]. In Ghana, Warm Heart Foundation (Ghana) Ltd. teaches biochar production from crop waste and tests biochar-based fertilizer with subsistence crops. In East Africa, Warm Heart Malawi Biochar Project has trained 3000 poor farmers, field workers from government ministries, veterinarians and nurses, and has established a multi-center biochar program in Kenya that also trains small farmers and field tests biochar [3]. We are reaching out to establish outposts in Uganda, Tanzania and Zambia. The African programs are linked by WhatsApp groups that share photos, videos, information, test data, and encouragement to the volunteers who manage them. The Biochar Africa and Biochar East Africa WhatsApp groups reach a wider audience including, we hope, field agents in Ministries of Agriculture.

How did Warm Heart arise? How did a retired professor of political science, a few volunteers and a small, untrained, local staff develop a new way of thinking about biochar and spread it so widely? How did it find a low-cost, do-it-yourself way to address the first three, big SDGs: Poverty, Hunger and Health? Why do people learn so easily to replicate the Warm Heart method in such different places? Trial and error – with the emphasis on error.

This admittedly non-traditional article is a structured narrative of how Warm Heart learned to develop a workable solution to what was dubbed an insoluble problem. It is the story of how Warm Heart came to realize that the common understanding of the ‘smoke problem’ was wrong (as were the ‘solutions’ built on that understanding) and then to form a new understanding that permitted the development of a novel solution. It reports how the Warm Heart leadership, the Director (myself) in particular, learned all this by doing. We/I did not choose to learn this way. We would have preferred to follow the guidance others who had addressed crop waste smoke “from the weeds” with the same aim of large-scale replicability, but we at least could find no one else.^3^ We found a wealth of biochar related materials from the developed world, but most were irrelevant to the conditions we faced and some contained a profusion of inaccurate ‘common knowledge’ about the rural poor of the developing world. (It turned out that they are not hidebound, out-of-touch, mired in customary practices, lazy, stupid or irrational.) In the end, we learned what we had to know the hard way – by trial and error. We learned, as my late father used to say, that “truth comes in blows.” (A favorite expression of Prof. Paul R. Shafer.)

This structured narrative is about Warm Heart’s learning experience, not about how Warm Heart taught small farmers. Yes, Warm Heart’s work has involved training thousands of small farmers in Asia and Africa, but Warm Heart did the learning, because this is really a story about how to effect large-scale behavior change – the basic aim of all development projects. It is, therefore, the story or how Warm Heart learned experientially to motivate large numbers of small farmers to change their behavior by approaching them in a new way and providing them with new alternatives that made change more attractive than stasis.

What did Warm Heart learn? We learned that even immensely important issues, such as the smoke crisis, may themselves be only symptoms of deeper causes that, if not addressed, will make solving those deep problems impossible, leaving the noxious symptom unsolved, too. We learned that, despite the tremendous amount of knowledge and analytic power possessed by large, outside organizations, without a grassroots understanding of the needs, concerns and motivations of small farmers, programs to change their behavior are likely to fail. We learned that the rural poor are hardcore rationalists who will not comply with programs that threaten their livelihoods or do not provide tangible benefits. We learned that the humanity of the rural poor lies not in their romance, but in their agency. Bottom line: Getting to our readily replicable, biochar social enterprise solution to the global crop waste burning problem involved Warm Heart *learning*, not Warm Heart teaching. Regrettably, a critical lesson we learned is that rest of the world is so isolated from the experience of climate change that they are not ready to learn.

## What is learning by doing?

Learning by doing involves no teacher, authority or leader who provides theory and structure. Learning by doing may look like flailing around in the dark or the blind reinvention of the wheel to traditionalists who believe that students need to be led or at least channeled in the ‘correct direction’ or to those who (believe) they actually know how things ‘ought to be done’. Learning by doing, however, is not about the details of *what* is learned; learning by doing is about *learning how to learn*. Not that these distinctions would have helped. Warm Heart did not have the possibility of falling back on a sure handed, guiding teacher. To the best of our knowledge, no one else in the world has attempted what we were attempting. To be fair, it should be said that, since we started, we have been told repeatedly that we do not know what we are doing and ought to listen to experts. To be fair to us, we have yet to find ‘experts’ to lead us.

For Warm Heart learning by doing was necessarily active or experiential learning in the best sense – a process of discovery. Armed only with a sense of mission and a set of human-scale problems defined by the members of our community to solve, Warm Heart’s learning process involved finding our own way by observing, testing, trying and failing, by attempting to understand subjects, problems and ways to approach them – alone. Colloquially, experiential learning is like learning to ride a bike; it involves a lot of falling off and tears, not classroom hours.

## What is warm heart?

Warm Heart is a tiny, grassroots, community development organization with outsized ambitions. What makes Warm Heart unique is that Warm Heart starts with an assumption of ignorance (as in, we do not know what your problems are and we do not arrive with solutions to them already in hand) and practices a policy of ‘ask, don’t tell’ that makes us listen as villagers describe what they think their issues are and explain how they would solve them if they won the lottery. When we arrived, we listened for a year, then organized programs around the same four categories of community-identified problems we address today and started work on their solutions in collaboration with the community.

The novelty of Warm Heart’s approach to both problem identification and solution definition is best summed up by that phrase: ‘ask, don’t tell’ and the starting presumption of ignorance, not omniscient knowledge. (Anyone who has studied methods in Rural Sociology will recognize that this is the ‘right’ way to do things. As a respected colleague observed, however, this is the first thing to be forgotten once it is clear that open-ended grant proposals do not get funded and that no one publishes null findings.) [Dr. Shafer’s personal communication.]

The outsized ambition that drives Warm Heart is the hope that if we manage to understand the origins of our communities’ problems, motivations and desires, the solutions tried, screwed up and slowly refined at home will then work globally. The point is not that Warm Heart will have all the answers, but rather that, because Warm Heart has taken the time to listen and understand the rationality of poor people’s actions, it will be positioned to do so again, molding field-tested, not theoretical, solutions to new circumstances.

The Biochar Project exemplifies the Warm Heart approach to problems and to our world. The program results from months spent in fields trying to understand open field burning from a small farmer’s perspective and only from here in the dust and mud trying to develop an alternative better for farmers and the environment. As Director of Warm Heart, I love getting a circle of poor farmers in an African village to laugh when I describe their situation perfectly. “How do you know?” they ask. I say, “I’ve made lots of mistakes in other villages.”

People often ask why we do what we do for strangers, and why we live in a poor farming community and listen to uneducated nobodies. We cringe. At Warm Heart, we do not think that our behavior is abnormal. Rather, like Ben Franklin, we believe that if we do not hang together, we shall surely hang separately. This is our only planet and we are our own only neighbors. No ‘they’ exists to fix what we break, be it a person or the whole world. We believe in radical responsibility, not of the bomb-throwing sort, but of the “if not you, then who? If not me, then who?” sort. We recognize only one binding rule from the opening of the Universal Declaration of Human Rights, “All human beings are born free and equal in dignity and rights” and we reject all efforts to rank humans according to ascribed orders. Who are you to call my neighbors nobodies? What makes you superior? Think about it.

## What did warm heart need to learn by doing?

**Fig. 1:**
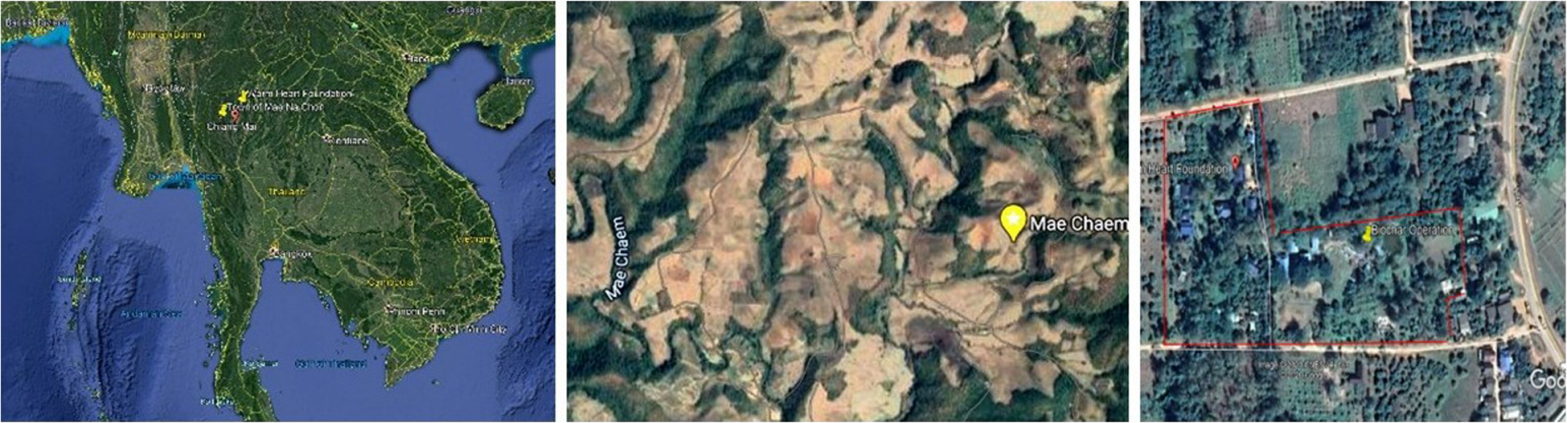
Left: Map locating the project site in N. Thailand; Center: The project site showing destruction wrought by corn production; Right: Warm Heart Foundation headquarters in Phrao, Thailand

Let’s start by placing this structured narrative in context, because the narrative, like Warm Heart’s learning, runs in a linear sequence (Fig. 1).

Our story begins with the smoke that cloaks North Thailand 3 months every year. This smoke, this ‘burning season’, causes an annual public uproar, spurs loud government pronouncements and ineffectual actions, and is then swiftly forgotten. Why? It is because most people believe that nothing can be done about the smoke or that any solution would be too costly or politically fraught to succeed. For everyone in Chiang Mai, the smoke itself is the issue.

After suffering through three burning seasons, Warm Heart decided that it was time to do something. We soon learned that it would also be necessary to do address the related public health, environmental and climate change problems. To confront the smoke issue, however, required, first, that we understand the motivations of the tens of thousands of small farmers whose annual field burning produces the smoke. Second, it required that we develop ‘technology’ so simple, so light, so cheap, so easy to fabricate that any small farmer could learn to make and use it him/herself. Finally, because farmers cannot afford to do this alone and outsiders will not pay them to do it, no matter what the long-term, global benefits, it required that we develop a sustainable business model that would make it more profitable to biochar than to burn.

While easily stated in retrospect, each of these three steps involved painful and costly learning for Warm Heart. We succeeded because we were willing and able to fail repeatedly until we got things right. We did not arrive with a single plan that we had the authority to impose (as might a government), nor did we have a carefully monitored budget for just a single iteration of a project before we had to quit. (As do NGOs with grants. Grant writers must declare victory before starting because they already know all the answers. Funders, after all, do not support grants that begin, “We think that ….” Project budgets, therefore, permit only one iteration and no re-dos. It is hard to be right the first time all the time.) Beginning from the presumption of ignorance, Warm Heart could be patient. We tried and when we failed, tried again. We listened to the other stakeholders in whose eyes we had fallen short. It was humbling, but people actually respected us for sticking around. The approach eventually succeeded, although the fundraising burden proved immense and over the years took its toll on our other programs and us personally.

## What is the problem?

If you can define a problem, you can solve it. Here, however, the big problem Warm Heart confronted – the big problem that long barred all solutions and stymied us, too – is that ‘the problem’ had never been defined correctly. It took a long time to recognize that the problem as defined was not the real problem, but instead simply a nasty symptom of the real problem. Insecure in our lack of expertise, we spent a lot of time thinking about the problem the way others did – only finally to discover that things did not work that way.

“What is the problem?” turned out to be a problem in itself. Where we live in Chiang Mai Province, ‘the problem’ is understood to be the smoke. Smoke is ***a*** big problem. For 3 months each year, smoke stops airplanes from landing, hospitalizes tens of thousands, kills thousands and costs Thailand billions. Like everyone, we started by assuming that smoke was ***the*** problem, or rather, that ***our*** smoke was the problem. However, ***our*** smoke is not unique. Our smoke is identical to the smoke suffered in China, Ghana, India, Iran, Kenya and Mexico.^4^ As long as we focused on *our* smoke, we failed to understand the bigger problem.

**Fig. 2:**
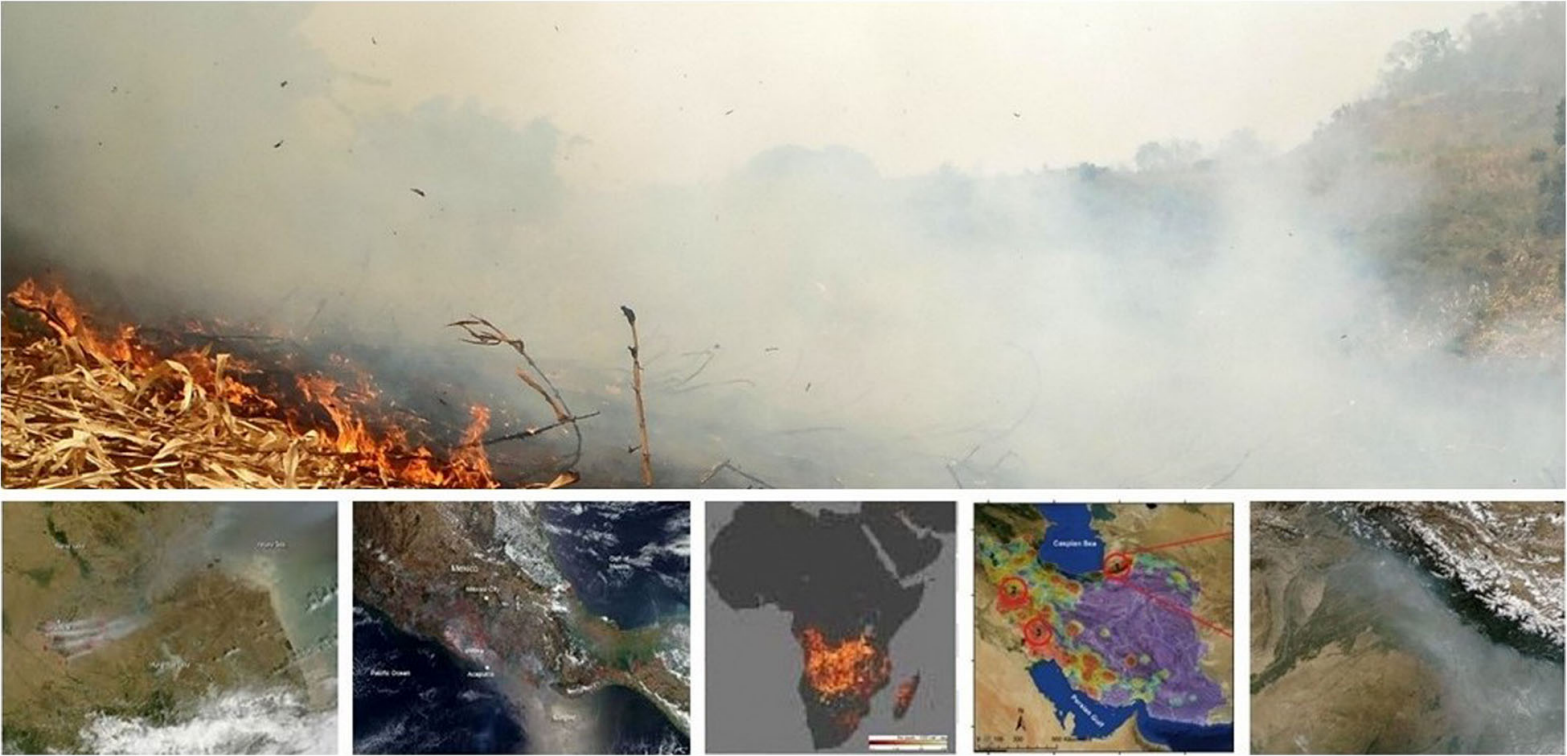
Our smoke; their smoke, same-same. Top: Burning corn field, North Thailand. Below: NASA MODIS satellite imagery of agricultural fires in China, Mexico, Africa, Iran, and the Punjab (India and Pakistan)

As is often the case, ‘learning’ that our smoke is not unique, but rather is typical, merely an example of a bigger problem, resulted not from massive data crunching or deep research, but from a moment of insight. Standing in front of a screen full of NASA MODIS satellite images of crop waste fire smoke from locations around the world, it was suddenly obvious that Chiang Mai, Delhi and Lahore shared the same problem that required a similar fix. The lesson was that Northern Thailand is not special; it is typical (Fig. 2).

**Fig. 3:**
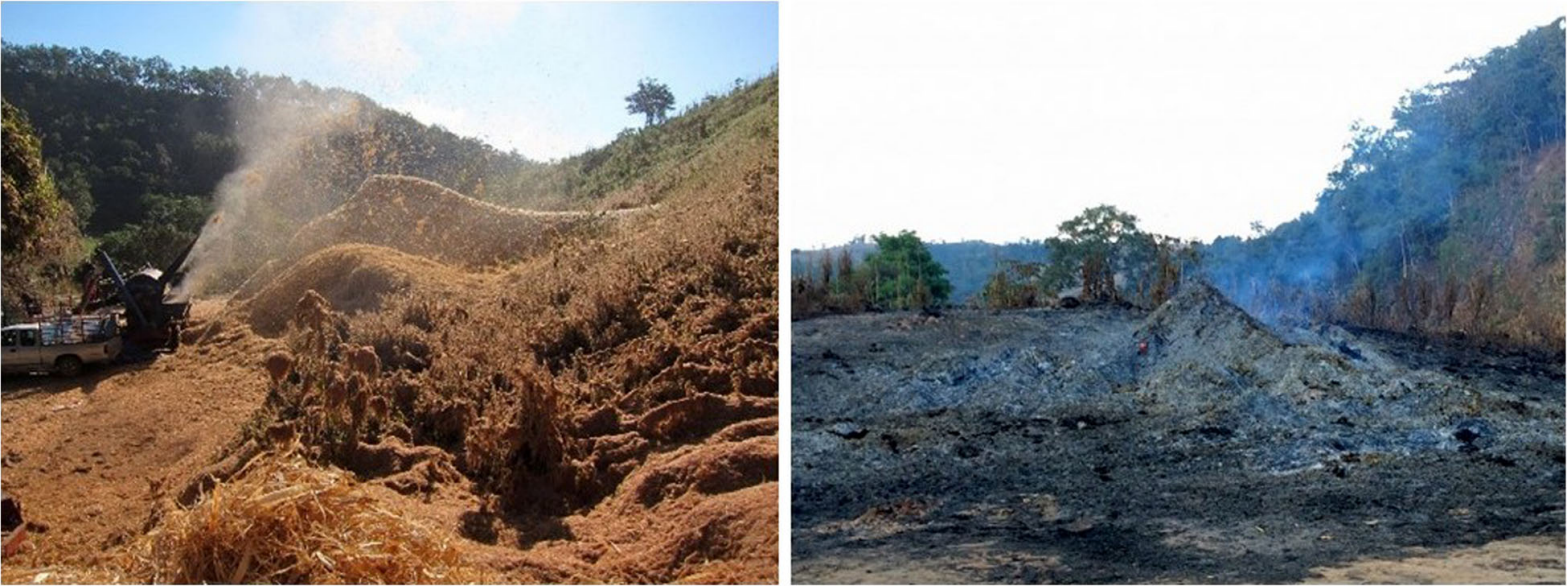
This (right) is what is left when you burn 1,000 tonnest of corn cob and husk (left). What you cannot see are the 1,585 tonnest of CO2, 1,073 tonnest of CO2e and 6.26 tonnest of PM2.5 released into the atmosphere

Our smoke, like the smoke plaguing the rest of the developing world, results from the open-field burning of crop waste.^5^ Crop waste burning is not an issue in the developed world. It is, indeed, hard to imagine a German farmer firing his rape field after harvesting.) Burning, however, is endemic in the developing world. Farmers here grow ten billion metric tonnes (tonnes) of food crops annually ([4] See Attachments 1–3). These result in 21 billion tonnes of crop waste. (The term *waste* refers to residues in fields too steep or with soils too hard to be tilled in and used by farmers too old or too malnourished to chop or collect the residues in the heat of the hot season, residues such as corn that are so difficult to decompose that the bacterial demand for nitrogen can drain the soil of this precious nutrient and/or residues in the fields of farmers without domestic animals to graze on them. In most cases, farmers also do not own the land or have reason to care for it.)^6^. Assertions about how much of this 10.5 billion tonnes farmers burn range from 50 to 90% and surely vary widely by location. If, on average, they burn just 50% – rounding down to 10 billion tonnes – these fires generate 16.6 billion tonnes of CO_2_, 9.8 billion tonnes of CO_2_ equivalent (CO_2_e) and 66 million tonnes of PM2.5 [5, 6]. Put in perspective, 26.4 billion tonnes of CO_2_ and CO_2_e are equivalent to the annual emissions of over 5.5 billion automobiles driven for a year [7]. Sixty six million tonnes of PM2.5 are equivalent to the smoke of 4,714,314,000,000,000 cigarettes. [Author’s calculation based on industry figure of 14 micrograms of smoke per cigarette or the smoke of 71,429 cigarettes per kg of PM 2.5.] According to the World Health Organization (WHO), PM2.5 is the fifth biggest killer in the world. PM2.5 kills 4.2 million people annually, 90% in the developing world, more people than are killed annually by the well-publicized infectious diseases – dengue, hepatitis-A, HIV, malaria and TB – ***combined*** [8] (Fig. 3).

If the issue is smoke, then we confront two problems. First, it is essential to know where the smoke come from and who is making it? These are obvious from satellite images.

The second problem involves figuring out why these people burn and so what to do. How Warm Heart addressed these questions and the answers we came to are perhaps the most important and controversial parts of this story. Addressing the why question demanded understanding the logic of current NGO and government policies, recognizing that this logic is wrong and proposing a new logic. Warm Heart’s critical ‘learning step’ involved a shift in perspective, from viewing small farmer, crop waste burning from the outside looking in, to viewing it from the inside facing out. We changed perspectives because we went out into farmers’ fields to work, then went home to eat dinner and drink white whiskey on the floors of their homes. Experts do not do that. As a result, most experts know that poor small farmers burn, but assume that burning is discretionary. The experts generally believe that small farmers burn not out of necessity, but rather burn out of ignorance, laziness, customary practice or simple orneriness.

The common misunderstanding that farmers do not *have* to burn but burn for other, *‘unnecessary’* reasons drives the two most common, most misled varieties of so-called solutions. The benign version, based on the notion that small farmers burn out of ignorance, cultural beliefs or outdated agricultural practices (inaccurately labeled ‘slash-and-burn’), argues that farmers need to be educated about how burning hurts the climate, environment and public health.^7^ The less benign version (seemingly embraced by governments everywhere), sees farmers as incorrigible lawbreakers who should be fined, jailed or shot.

Understood from the perspective of a small farmer, burning is not discretionary; burning is necessary. Poor farmers have fields often located on challenging terrain. They are too poor to afford tractors and most fields are not tractor accessible or too rocky and steep to plow. Most crops leave at least as much waste in the field as they provide edible produce, many much more. (Corn, for example, is 63% stalk, 11.1% cob, 3.7% husk – 77.8% waste and 22.2% kernel.) [Data from Mae Chaem District Agricultural Officer and farmers’ rule of thumb.] To prepare to plant, a farmer must clear the field of waste and accumulated weeds; given difficult terrain, lack of labor and heat, burning is the only option. NGOs may educate farmers about climate change and PM2.5, and governments may threaten them with fines, jail or death by sniper – but they still burn because they have no better option.

Warm Heart’s first real challenge was to define the ‘smoke problem’ correctly. Getting it right did not require new technology, data or analysis. It required only a change of perspective born of talking to small farmers and listening to what they said. The ‘problem’ was not the smoke, the accompanying climate change gas emissions or the public health damage caused by PM2.5, as everyone else argued; these were *consequences*. The *problem* was small farmers’ lack of alternatives when confronted with the necessity of clearing their fields of previous crop waste and weeds.

It took Warm Heart years to recognize the ‘problem with the problem’ problem. Smoke was so obvious. What did we know? When we figured it out, everyone told us that we were wrong, that we did not understand smoke in general or these little creeps, in particular, who are best stopped by a bit of good, tactical shooting to stop the fires dead, so to speak.

## Developing truly “stupid technology”

**Fig. 4:**
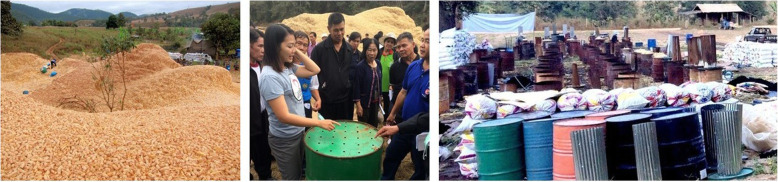
Left: For scale, granny collecting cob on pile; Center: Aom, Warm Heart’s Biochar Project Manager teaching farmers to make a TLUD; Right: TLUDs, extra barrels and piles of bagged char at test site

If you follow biochar posts on Facebook or LinkedIn, biochar blogs and so on, you know that biochar is a First World thing involving technologies beyond the reach of poor farmers.^8^ The first step we confronted was thus to develop biochar-making technology so cheap, easy to make, use and transport that any poor farmer anywhere could clear his/her fields with it. If any place existed where we thought that reference materials would be available, this was it. We were wrong. In fact, we could not find technology that met our specifications.^9^ It also proved amazingly difficult to design what we fondly call ‘stupid technology’ as in: KISS, “**K**eep **I**t **S**imple, **Stupid**,” a favorite expression among the design team (Fig. 4).

We started with John Rodgers’ TLUD – the **T**op **L**it **U**p-**D**raft biochar machine,^10^ the famous JRo (or Jolly Rodger). It was almost perfect; we just made it cheaper to make and easier to move.^11^ Farmers, however, complained that the JRo is too small to make much char from common low density feed stocks (e.g., rice straw) and too hard to load with others that do not fit neatly into a barrel (e.g., tree branches). We tried increasing the volume. The resulting FU2 (frustrated, us?) and FU3 increased volume to three and nine cubic meters respectively. They produced great char, but farmers hated them. They took too long to load and empty, and weighed too much to move. Total failure and much money wasted.

Technologies must be acceptable to farmers and good for a variety of feed stocks. The TLUD was ideal for chunky stuff like corncob, but could not handle enough of the low density or awkward stuff to be an all-purpose machine.

Next was the inelegant trough that resembles a watering trough for cows. It is a steel box with slanted sides and can be as large or long as desired [10]. (We make 500- and 2000-l troughs, and a three-meter XL (extra-long) for bamboo. You can make it as you like and need it.) The trough allows no oxygen to enter from the bottom. The fire burns near the top, anything that drops below the flame falls into an oxygen free space where it remains as char. Small particulates burn in the flame above. From a farmer’s perspective, the trough is great because it permits charring of stalks and straws, branches and bamboo without stuffing or chopping (a nasty chore during the hot season). Cost, manufacturability and weight, however, count against the trough. Made from new materials, it is expensive; made from scrap, it is not. Either way, it requires a welder. Big troughs are heavy; farmers must leave them in place and bring feedstock to them. This works in orchards or where a farmer can rake stalk downhill; otherwise it is heavy work.

**Fig. 5:**
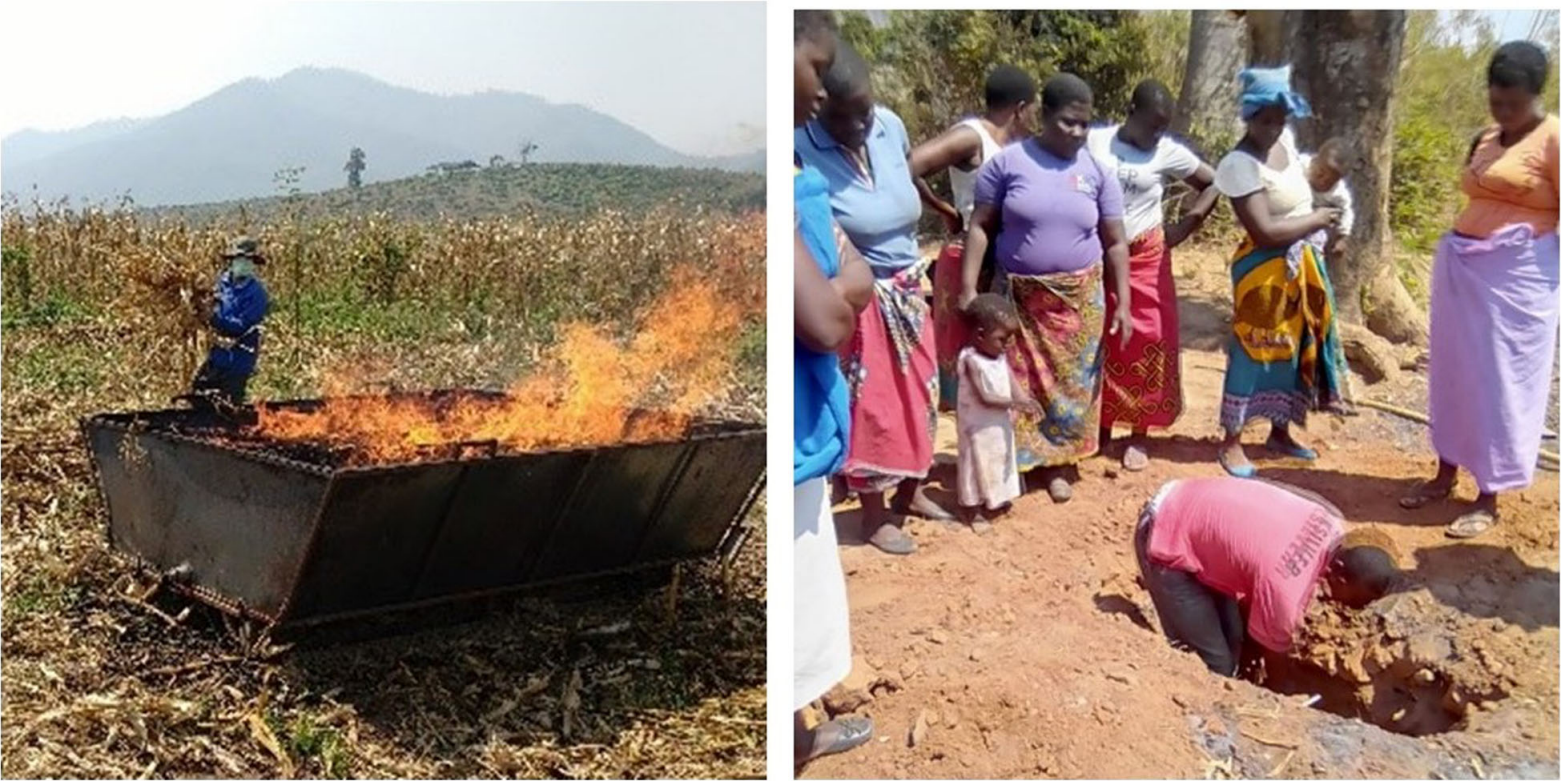
In wealthy Thailand, it is all about making money. Using a big trough (left), a team of three can make 6,000 litersl of corn field or tree prunings char a day. During the dry season when no work is available, each team member can make double the average local wage. In poorer Malawi, farmers keep all the char they make in a trench (right) for fertilizer

When Warm Heart began operations in Africa, cost made the trough unviable. The solution proved simple, cheap – and all but eliminated the trough: dig a hole in the ground. If a trough is a box that contains the fire and excludes oxygen; a trench is a ‘trough’ in the earth. The trench’s benefit is that it requires no money – no steel, no welder, no nothing. You do not have to move it. Every farmer has a hoe and if a new trench is required, it is a matter of minutes to provide one (Fig. 5).

Then an unexpected problem arose. Because we test at home, we did not anticipate farmers’ biggest problem: quenching the hot biochar. We use a hose; poor farmers do not have water in their fields. (Yeah, I know, obvious, but then, there it is.) Without water, you must smother – but this poses problems for each technology and takes hours. Smothering a TLUD requires moving the hot machine and setting it deep in dust to block the air holes at the bottom. You must then remove the stack assembly to apply a sealable top. These are available, but the barrels they come with are costly. Smothering a trough is worse. Hot troughs distort, making tops hard to fit and requiring a homemade ‘fire blanket’ of thick canvas soaked in water to lay on top. Watched carefully by someone with a few buckets of water (hand carried to the field) to stop it from burning through in spots, the fire blanket will extinguish a trough quite fast. (Before mastering the technique, however, you burn many holes in the ersatz ‘fire blanket’ and patch lots of canvas.) Trenches are easy. If you dig a trench slightly smaller than a sheet of corrugated roofing tin, you can slide the sheet over the trench. You then pack the dirt removed when making the trench around the edges of the sheet. In Africa, poor farmers cannot afford roofing tin. This inspired the perfect, zero-cost, biochar, field ‘stupid technology’ – a hand dug trench extinguished by kicking in dirt [11].

We must be honest about this. If Warm Heart knew what it was doing; If Warm Heart was staffed by certified and accredited professionals; If Warm Heart had knowledge resources on which to draw; If Warm Heart had money. If, if, if …, then this entire, lengthy and frustrating low-rent technology development process would not have been necessary. If we could have read an article or perhaps some smart guy could have come along and sorted us …. Warm Heart, however, is a typical small NGO. It is under-capitalized, under-staffed and under-certified. We worked hard, did our best, but it still took us 3 years of learning by doing to develop a solution to the global crop waste burning problem. If help was available, we did not know where to find it. Had it been offered, we would have accepted. As it was, we learned the hard way, by often falling off the proverbial bike.

## Achieving self-sufficiency

The hardest lesson to learn had nothing to do with smoke; rather, it involved the shock of learning that no one seemed to value what we were doing. We had a ‘stop-the-smoke’ solution that removed CO_2_ from the atmosphere, reduced CO_2_e emissions, cut smog precursor and PM2.5 emissions and improved public health – but we could find no one to support us. Initially, we believed that the problem was our focus on smoke, an issue that lacked traction with the global public. We, therefore, researched the connections between crop waste burning and climate change, deforestation, habitat and biodiversity loss, watershed destruction, loss of food security and so on. No use.

We read constantly about high level meetings that promised funds for carbon removed from the atmosphere and for the development of new carbon removal systems. We read about the climate crisis everywhere, as well as about falling food security, the impact of climate change in the developing world and rising fear of hunger migration North. (Some EU countries apparently now condition grants to Africa on the likelihood that projects will stop migration.) In the end, however, to get help, we had to win grants or places in business startup incubators in California. We failed to win the big grants or spots at boot camps.

After months of Warm Heart complaining about the cruel world, a senior international organization official had the courtesy to tell us that no one would look at our project until it was profitable (Dr. Shafer’s personal communication with the President of an FAO subsidiary for the Asia-Pacific region.). This became the third and biggest challenge we faced. Could we transform a development project for the poorest of the poor that would cool the climate, clean the environment and improve public health into a profitable business that would also interest real investors?

Here Warm Heart confronted both a serious experiential learning challenge and perhaps the starkest possible example of what is wrong with most of the so-called ‘sustainability’ movement. What to do?

True, you will find many stories of successful entrepreneurs who have turned plastic recycling by street people and the distribution of uneaten restaurant meals into profitable businesses. The devil, however, is in the details and winnowing out the winning details blind folded, so to speak, proved harder than you might think. I tried my best, but I could not craft a ‘value proposition’ or ‘unique selling point’ that I felt I could defend on Shark Tank. Maybe it is just me. Maybe I am the problem here. Maybe if I was someone, anyone else, I would have had no trouble converting the Warm Heart Biochar Project into a successful startup – but I doubt it. I may be wrong, but I do not think that everyone who has built an NGO is necessarily prepared to launch a startup.

Interestingly, no one offered useful advice about how to make it in the market, but everyone had ‘sustainability’ advice, much of it vacuous. Most advice began (and ended) with the notion that just ‘doing good’ is enough. This is not true. It is not enough to ‘be green’, consume without adding to your carbon footprint, take only bicycle vacations or whatever. These do not help reverse climate change. They are also not sustainable either in the old sense of the term (“able to be maintained at a certain rate or level”) or in its more modern sense (“conserving an ecological balance by avoiding depletion of natural resources”).^12^ Climate and environmental problems are systemic and require ongoing responses that can maintain themselves without subsidy, support or other intervention.

What we learned is critical and probably could only have been learned the hard way. Our shocking realization ought ***not to*** have shocked us at all. Can you name a cause that people will pour money into *forever*? No. This is why the charity model of environmental remediation fails and why politicians and policy makers shy away from the financial black hole that is ‘fixing’ the environment.

What Warm Heart learned the hard way, what today makes the our model unique, is that over the long-term, across large and diverse populations, altruism and calls for social solidarity to ‘save the earth’ simply do not work. What works is the personal profit motive and disinterested, ‘a-human’ action of a market, especially when offered to those who have been *excluded from opportunity before*. Now, *this* was a shocking lesson for someone like me born into the communitarian tradition of environmentalism. Once I/we learned it, however, and since we have put it into action, poor farmers making biochar have flourished and, *entirely incidentally*, CO_2_ has been sequestered, CO_2_e, black carbon, smog precursor and PM2.5 emissions have declined, public health has improved, and rural poverty has fallen. Moreover, this will be sustained until small farmers and/or their clients are provided a still better alternative. I do not know when it finally occurred to me that we were running a market access project for the long excluded rather than an environmental project, but calling the moment ‘enlightenment’ might just work.^13^

## A social enterprise solution: learning to let the market do the heavy lifting

Social enterprises convert social problems into profitable business opportunities that benefit all stakeholders, most notably the excluded poor, best without inciting the ire of the powerful.^14^ Social enterprises have three bottom lines:

1. *They must be profitable*. If they are not, they die. Profitability is not part of the common definition of ‘sustainability,’ but must be. Social enterprises cannot depend on outside funding because all funding ends when funders lose interest -- and all funders lose interest, taxpayers sooner than most.
2. *They must solve the social problem on an ongoing basis* or they are flash-in-the-pan failures. Real social problems result from deep, underlying conditions that require constant attention. If the means to address them is not permanent, they will reemerge.
3. *They must benefit all stakeholders*. If they create or exacerbate other social problems, leaving the locale no better off than it was to start, they fail.

As a grassroots community development organization serving the rural poor by partnering with communities to solve villager-identified needs, Warm Heart takes these three requirements to heart and understands why so few real social enterprises exist. (Note, because it is cool, many organizations falsely self-nominate as social enterprises and universities even claim to convert students into social entrepreneurs, but buyer beware.)

When defined properly, a social enterprise is, in effect, a small business distinguished by a bottom line that includes considerations of community, environmental, economic and social benefits. As a grassroots NGO, however, Warm Heart had no clue how to build a small business. The number of mistakes we made would fill a book. To summarize, we faced three clusters of problems. (1) How to organize the prospective social enterprise. (2) How to engage small farmers in it. (3) How to create a market for its products.

## Organize

**Fig. 6:**
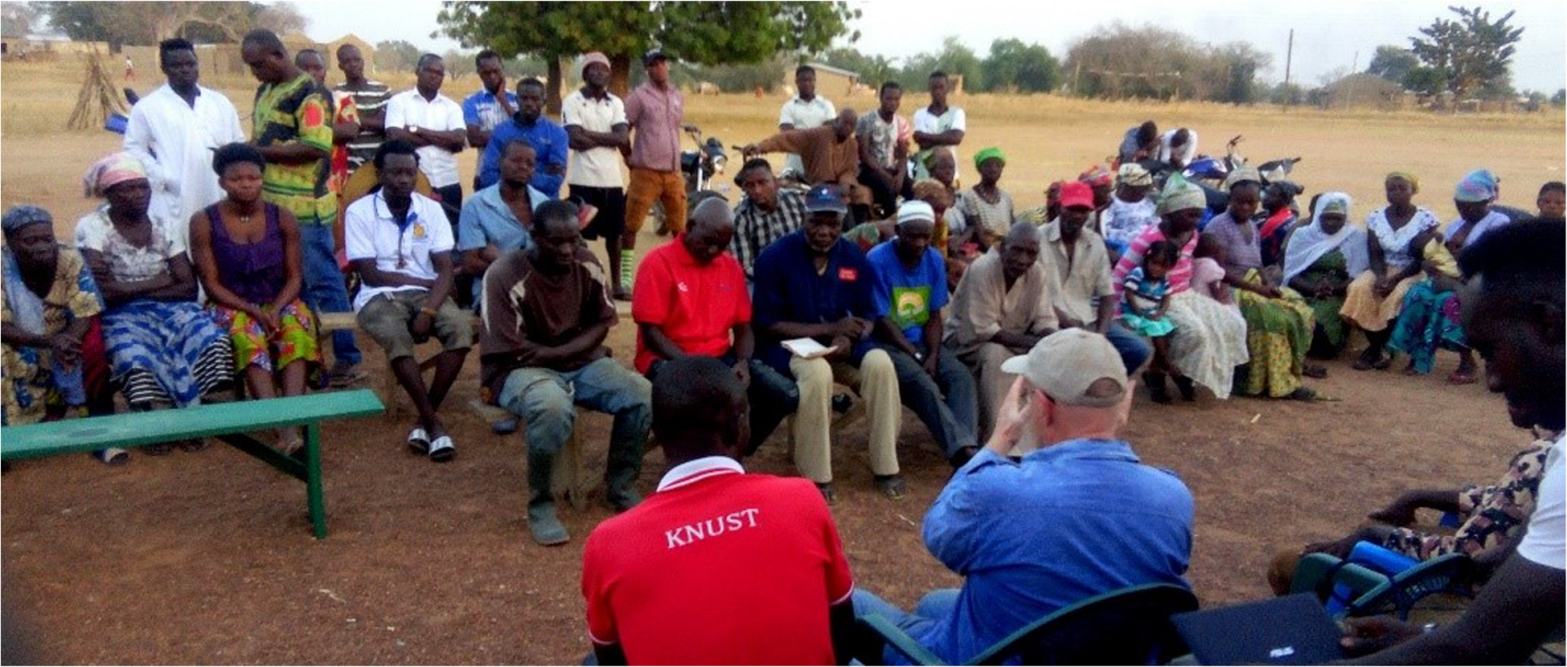
First meeting of the CAC in Nganga, Upper North East, Ghana. Seated in front, the chiefs, standing in back, the senior farmers, seated to the right, the ‘shadow cabinet’ of senior women

Warm Heart begins all projects by forming a Community Advisory Committee (CAC) and did so here, too.^15^ In this case, the CAC debated proposals and vetoed many, including the idea that Warm Heart or any outside organization would play an ongoing role. The CAC insisted that the social enterprise be autonomous, owned and operated exclusively by working members. (Learning by doing is universally daunting. The CAC would rue this decision, but how was it to know at the start?) (Fig. 6).

The final model for the first social enterprise called for an internal division of labor. Certain farmers would convert crop waste into biochar; others would remain in the village to process the biochar into value-added products. Everyone would be paid for his or her work; farmers for a set number of days per tonne of biochar; those working in the village for days worked. The coop would sell products to an outside broker. The fiscal year would end during the rains, after production ended. All coop members (villagers who worked during the year) would attend a final meeting to vote on the division of profits between the next year’s operations and members. Our hope was that coop members’ success would lead other villagers to join, further reducing burning, increasing the biochar supply and growing the business.

## Engage

From the start, most people warned Warm Heart that farmers would not make biochar. The first experiment we conducted (now several years ago), therefore, tested this by offering villagers a small price per kg for biochar. Before we began this experiment, the CAC told us that if we wanted farmers’ cooperation, we also had to live in the community throughout, visibly there to help if anything went wrong, and promise to buy every kg of biochar produced (because “we are farmers, not salesmen”).^16^ We rented a house in the village, have kept a local presence for 3 years now and agreed to buy every kg. That first year, farmers made 15,000 bags of biochar and every Friday we paid cash on the barrelhead. Today, we hold a formal meeting with the CAC a month before the harvest starts where we provide an estimate of how much biochar we can afford to buy during the coming dry season. (This year, with Covid refugees swelling the ranks of the unemployed, biochar making is the only local source of income is some villages.)

A month after we began production that first year, the Government tried to imitate us, but made no effort to engage the community. They arrived one morning in the neighboring sub-district, ordered everyone to attend, provided a thorough training (actually using some of our training materials) – and left villagers to find markets, organize transportation, etc. The sub-district produced ***not one*** kg of biochar. Between our success and the Government’s failure, we felt that we had a natural experiment testing the value of “ask, don’t tell”, depending on a CAC and engaging community members by listening. In fact, this incident provided one of the few pieces of concrete feedback during our learning process that we were on the right track.

How do we know we succeeded? Or, to put it differently, what would success look like. On site, we believe we succeeded because, farmers continued to make biochar and to talk to us, while they did not make char for the government and spoke to government officials only when spoken to. On the other hand, while Warm Heart succeeded in the field, we faced and continue to face a financial crisis, today only exacerbated by Covid-19. This brings us to the biggest business problem of all: creating a market.

## Create a market

It is easy to understand the value of not burning, making biochar, reducing emissions, and so on – it is another to sell biochar. Here Warm Heart suffered from our own naïveté. We considered biochar-based fertilizers the best option. Our soils are terrible; biochar mixed with organic matter provides an ideal fit. When buried in the ground, biochar also sequesters a large quantity of carbon (C) extracted from the atmosphere by photosynthesis. Thailand consumes hundreds of thousands of tonnes of imported, synthetic fertilizer annually. Now it wants to go organic. We, therefore, (innocently) thought that a potential agricultural market existed. Wrong. The biggest corporations in the business dominate the fertilizer market and their products are available, like Coke, in the remotest villages. (In the areas where we work, farmers report on surveys that fertilizer and seed salesmen are their most important source of information.) What of the booming international market for biochar? We cannot match the prices or volumes. Why? If, for example, you own a lumber operation that burned saw dust to kiln dry boards, you can use the heat from your biochar operation instead, capture valuable wood vinegar, get the biochar as a free byproduct and load it into readily available containers to meet orders for 200 t a month. We do not want to run a race to the bottom with operations that produce costless tonnes of biochar and could never hope to compete at this scale (besides, how do you get a container into the mountains?). Nothing in it for farmers.^17^ What to do?

*Briquettes*

Because Warm Heart is committed to reversing climate change, it hurt to accept that producing biochar cooking briquettes could save Warm Heart. Burning biochar releases into the atmosphere the C that biochar can potentially sequester. Bad, but we could see environmental, public health and market reasons for making and selling biochar briquettes. Sixty five percent of northern Thais cook with charcoal**.** (In 2017, UNData estimates that the developing world consumes some 59-60 million tonnes of charcoal annually. A link to the UNData database can be found following this article in the section entitled “Availability of data and materials.”) Charcoal is nasty stuff. It smokes and emits noxious gases when it is made, and wastes most of the wood used. (Efficiency is just 7% in country kilns suggesting that 60 million tonnes of charcoal requires 857 million tonnes of forest wood to make.) When burned at home, charcoal smokes terribly and emits more noxious gases, contributing to many deaths from indoor smoke [12]. Charcoal is also charred wood, which makes cooking on charcoal the enemy of forests, habitats, biodiversity and watersheds. [Asserting that the situation is typical of all Asia, an FAO consultant reported long ago that in Iran, 4 million m^3^ of wood are made into just 400,000 m^3^ of charcoal annually, while only 200,000 m^3^ of wood are cut for other purposes [13]. Making biochar from crop waste produces no smoke and requires no wood. Crop waste biochar disconnects cooking from forest, habitat, biodiversity, and watershed destruction. (Some argue that biochar briquettes spell the end of forests. On the contrary, North Thailand currently produces almost one million tonnes of corn waste alone annually, enough to make at least 200,000 t of biochar briquettes or 200 kg per charcoal burning household.) [Author’s calculations assuming 20% efficiency, 4 persons per household and a population of 6.3 million. 7 Before switching to hotter and longer burning biochar briquettes, Warm Heart used 60–80 kg of charcoal per week to feed 60 people (fifteen households) a day, the equivalent of 208 to 277 kg per household per year. N. Thailand also produces millions of tonnes of burned rice straw and orchard tree prunings, as well of millions of tonnes of vegetable tops, potato hay and so on.]

When burned, biochar does not smoke or emit noxious gases. This cuts the threat posed by indoor smoke, estimated to kill some three million people cooking with biomass per year [14]. Clean stoves, on the other hand, are too expensive for most of the global poor and still require wood, even if less than before. As for the market, biochar briquettes look exactly like the already accepted charcoal briquette and sell for the same price. In rural areas, they also sell into a market protected by its small scale and fragmentation, which keep big sellers from entering and driving down prices. Warm Heart and other local brokers, therefore, can sell on the briquettes that they buy from social enterprises, making the reliable market that villagers require.

Our decision to make and sell biochar briquettes has not endeared us to everyone. Many in the developed world biochar community are offended by the very idea of burning “bio” char, the sole purpose which, in their minds, is to improve agricultural outcomes. More consequentially, the ‘clean stove’ lobby (led by the Global Alliance for Clean Cookstoves but including many other, lesser organizations) is big, powerful, well financed and backed by everyone from Microsoft to the World Bank, objects strenuously. Our only objection to the Alliance’s work is that they do not have a ‘CAC’ equivalent such that we have not yet found an area in which ‘clean stoves’ are readily available on the market for prices anyone can afford and do not require wood. Individual ‘clean stove heroes’ such as Paul Anderson in India, Kevin McLean in Africa and Paul Oliver in Vietnam have reached hundreds of thousands of households, but they are few and far between. Today, three billion people still burn biomass – too often charcoal, dung (that could be used as fertilizer) or wood – while the total number of clean stove users is still measured in just the hundreds of millions.

## Conclusion: can learning take you only so far?

This has sounded like a fairy story as all barriers have fallen to Warm Heart’s commitment, caring, creativity and patience driven experiential learning process. Wrong. Warm Heart is in a real bind and this is what I want to leave you with. We really do not know what to do. When we opened our doors in 2008, we had just an idea, a way of thinking about how to be an NGO. Today, in 2020, we are thriving in our community, but financially stressed to the limit in the “real world” of NGOs. Why? Two reasons. First, our development model does not sell. We are failing at fundraising in the places where it matters, which is not in the villages. Because we respond to community-identified needs, we cannot do as most other NGOs do when fundraising: sell a terrible problem and a clear, straight forward solution to it. Our presumption of ignorance, asking locals, multiple projects to meet the complexity of communities’ felt needs do not lend themselves to a short, sexy one-pager. Hey, no one has the time to listen to our litany. It is all just too much of too much. Second, we are still failing at what we know is our most important task: making ourselves self-supporting. (If you are really big, you can afford to spend 50% of more of your budget on fundraising and so not have to worry about making a profit on your core activities. Warm Heart depends on volunteers for its marketing and really ought to be able to monetize the value of the services it provides.) Here is where biochar comes in. Biochar is not simply about social enterprises for poor farm families; biochar is to generate a revenue stream above costs, real income for Warm Heart. Biochar has cost Warm Heart thousands and thousands of dollars, most stripped from basic operating budgets because we believed so strongly in what we were doing for the poorest farmers and the global climate, and because we could not find support elsewhere. This cannot go on. Biochar must begin to break even or even turn a profit, if not to pay back the other budgets, at least to remove the stress.

Warm Heart faces a real conundrum. We know now how to analyze the “smoke problem” and solve it. Indeed, we believe that we have a potentially globally replicable solution to an important, global climate and public health problem that can help reduce rural poverty. We have also learned, however, that we may have limits to our own capacity as an NGO to do what is necessary to grow what we know from “good idea” to viable, scalable, replicable commercial endeavor, perhaps even make it profitable enough to pay its own way. My final question for you to think about, therefore, is this: What do you do when experiential learning teaches you that you cannot do what you must?

Remember the Clinton campaign’s famous: “It’s the economy, stupid”? Here ‘it’ is “What did the dummies at Warm Heart learn?” This is where I want to leave my story – with the question of whether the ultimate failure of Warm Heart to launch a profitable social business is due to ***our*** limits or is endemic among small, community development NGOs? Put more bluntly, the question is “Whose fault is it?” as in “Could another Director or NGO do better?”

Well, of course, someone else could have done better. Many people have the necessary talent to build a biochar market. That is not really the point, however, since we have little reason to believe that many such people would linger long in our world of marginal NGOs. More to the point is what kind of NGO would it take? And here, I honestly believe that small, community development organizations that begin by listening to people and build programing to meet their expressed needs are simply not made to launch successful businesses. They lack the skills, resources and access required to make projects of this sort into a globally replicable, scalable businesses. In fact, I find it hard to imagine how such an NGO could remain focused on the local when operating globally as a venture capitalist or investment banker.

So, what message should you take away from this lengthy narrative? I hope that you leave with four provocative ideas stuck in your head. I hope that I have made the value of experiential learning as a process of discovery clear enough that you will consider employing it. I hope that I have taught you enough about the value of getting down in the weeds that whenever presented with expert opinion you will always ask the only methodological question that matters: how do you know? I hope that I have sold you on the possibility of a replicable, low-cost, low-tech, “good enough biochar” solution to global crop waste burning well enough that you will help keep the idea alive. Finally, I hope that if you head a small, grassroots NGO, I have demonstrated that while you may face endless barriers and grief from those who “know better,” *you should not give up*. You may not become a Silicon Valley sensation, but you can grow good ideas that others bring to fruition. Remember: if you live for the money and glory, you would not be stuck here in the public domain. So as my old man used to tell me, “Pull up your socks, kid, and go change the world.” (Another Prof. Paul R. Shafer phrase, often stated somewhat more colorfully.)

## Supplementary information

**Additional file 1.**

**Additional file 2.**

**Additional file 3.**

## Data Availability

All data and other materials supporting the conclusions of this article are publically available or are attached to the article itself. The datasets supporting the conclusions of this article are available from the Food and Agriculture Organization’s statistical branch, FAOSTAT, http://www.fao.org/faostat/en/#data, NSO, the Thai National Statistical Office, http://web.nso.go.th/en/ and UN Data, the United Nation’s open source collection of databases, http://data.un.org A dataset and its analysis that support the conclusions of this article can be found within this article as Additional files 1, 2 and 3. Additional files 1, 2 and 3 submitted as Word files with data included in tables. Additional file 1 (Attachment 1), entitled “List of developing world countries included in calculations.” lists the countries included in calculations and explains the reasons for country inclusions and exclusions. Additional file 2 (Attachment 2), entitled “Crop production to waste ratios,” explains the field waste to edible crop ratios for different types of crop. Additional file 3 (Attachment 3), entitled “Crop waste, emissions from crop waste burning, and impacts,” combines the data from Additional files 1 and 2 with Emissions Factors from S. Akagi et al. [15], to calculate the emissions generated by the open field burning of the identified crop wastes.

## Footnotes

1 “Biochar” per se is not a fertilizer. It is as sterile as a surgeon’s scalpel when new. It is technically a “soil amendment.” That said, at Warm Heart, we never put plain biochar in the ground; we mix it with manure, urine and EM making, at least in our eyes, a fertilizer.

2 Mention of “briquettes” will raise hackles; given their role in this story, let me address them here. “Bio” char is normally defined as “life supporting,” which rules out burning. Focused on climate, many also find talk of briquettes distasteful. Warm Heart, however, needed product to sell; the stuff that goes in the ground does not sell and briquettes do. How to justify briquettes? Biochar burns clean. No methane, no NOx, no PM2.5. It is not as good as an excellent clean stove, but such stoves are a decade away in most places. How many people need to die waiting? And nomenclature? Why make life more difficult? I avoid the fancy names for biochar machines because if it makes biochar, I do not care what it is called. Biochar? Char? Whatever.

3 Later, we learned, for example, of Hans Pieter-Schmidt’s work in Nepal, but have subsequently realized that the primary problems that we confronted lie between the intense, small- (village-) scale engagement of his work and development of a model that would replicate to scale. More generally, we have yet to find, perhaps because of our own isolation, anyone else engaged in replicable, field-level stop the burning or biochar-based development work. At issue is the necessary focus on large-scale behavior change. There are many excellent field technologies for small farmers; what we have not found yet is another systematic inclusion of such technology in a sustainable, readily replicable model. We believe that it is our effort to connect our field work with success in large-scale behavior change that sets us apart.

4 This story excludes the smoke suffered by Singapore and Malaysia as a result of forest burning to clear land for palm oil plantations in Indonesia, Borneo and New Guinea. It does because each fire is a onetime occurrence. There is a lot of forest to burn. Once burned, however, the land is protected because it is plantation. Crop waste burning is annual.

5 In North Thailand, the loudest complaints about smoke come from Chiang Mai, a city surrounded by forested mountains. Residents blame forest fires – irrelevant elsewhere – for smoke. High-resolution satellite photos (not free on the internet) reveal, however, that most forest “hot spots” originate at small corn farms in the woods. [2, Dr. Shafer’s personal communication with Mae Chaem farmers.]

6 Understanding the crop waste problem required learning that does not need to be detailed here. In brief, few researchers account for the excess of waste over edible crop, out migration of able bodied adults from rural areas, malnourishment or the soil chemistry consequences of plowing in certain wastes.

7 We love the “stupid traditional farmer” version. The government complemented a place where we work for showing no “hot spots” on satellite images, the gold standard of burning evidence, although a study proved that 41% of corn stalk had burned [9]. When we asked farmers how this could be, they told us, “We know not to burn when the satellite goes over.” [Dr. Shafer’s and Warm Heart staff’s personal conversations with farmers].

8 At a training 2 years ago, the senior agronomist of a plantation told me that he had never considered biochar because the first words he heard from consultants were always “millions of dollars.” [Dr. Shafer’s personal conversation.]

9 Many polished field machines exist such as the Kon-Tiki, as well as rough and ready ones made of sheet metal. We reviewed them all, but farmers resisted. “Too heavy,” “too awkward,” “too expensive,” “can’t make it,” “can’t move it,” they said. They also refused FU3, the most expensive machine we ever built, now used to make compost.

10 I use “biochar machine” to avoid getting into the “what-to-call-it” battle in the biochar world.

11 The original used three barrels, making it prohibitively expensive. By eliminating two and switching to cheap sheet metal for the stack, we cut the cost 75%. By shortening the stack, we made it neatly packable for easy transportation. Not exactly rocket science, but then, this is the world of used oil drums.

12 This very definition is problematic. Any process that produces waste ***anywhere*** is consuming limited resources. There is no such thing as strip mining “just enough” copper if the required energy is not entirely recaptured, the overburden and chemicals used to extract the copper not reused, the copper itself not recycled 100%, etc. We can do better than we are, but the notion that “reduction” as in reducing the amount of carbon added to the atmosphere will save us is not sustainable.

13 Reference to the “excluded” is critical. The village rich and powerful do not participate in these projects, the poor do. They do because biochar making lies outside of the controls that bound them before, because through biochar they have access to a market where their work is rewarded proportionately.

14 The importance of categorizing projects was brought home recently when a staff member stopped me mid-sentence to say that providing villagers air-tight storage units to hold their corn until the price went up was dangerous. “Why?” I asked. “Our other work creates new money; this project will take money away from the most powerful man in the district. Do it only if you are ready to fight,” she said.

15 Out of respect for village structure, CAC development always begins with the “elders” who are wealthier and more powerful. Follow on meetings generally move to all-village settings and the big boys and girls stop coming. The defining members of projects are therefore usually the poor and marginal for whom biochar is a blessing.

16 Villagers told us that if we did not agree, they would not agree because they were tired of being treated as if they were invisible by government and NGO “experts.” [Personal communication to Dr. Shafer and Warm Heart staff.]

17 One can imagine a scenario in which the countries of Southeast Asia discover the value of biochar-based fertilizers, water filtration and decontamination. In this event, demand might push supply enough to raise prices. The risk here is that large suppliers will meet higher demand by cutting forests and providing supply through existing supply chains, excluding farmers.
