# Supplementary material for: Global crop waste burning – micro-biochar; how a small community development organization learned experientially to address a huge problem one tiny field at a time
Source: Sustain Earth. 2020 Nov 23;3(1):18. doi: 10.1186/s42055-020-00037-y (PMC7680978; doi:10.1186/s42055-020-00037-y)
Supplement: Supplementary file 1 — Additional file 1. [file 42055_2020_37_MOESM1_ESM.docx]

**Attachments 1-3**

All data included in Attachments 1-3 can be found at <http://www.fao.org/faostat/en/#data>.

**Attachment 1: List of developing world countries included in calculations.**

Ignored are all small population and/or developing oil states (e.g., Saudi Arabia) and/or all American, English and French overseas dominions (e.g., the Solomons, Bahamas, New Caledonia) and/or micro-states and economies (e.g., Djibouti, Niue), and Russia (although a developing country and heavily agricultural). There are several surprising inclusions. These involved banana, coconut or sugar cane production that broke 50,000 tonnes (e.g., Kirabati).

| Afghanistan |
| --- |
| Albania |
| Algeria |
| Angola |
| Argentina |
| Armenia |
| Azerbaijan |
| Bangladesh |
| Barbados |
| Belarus |
| Belize |
| Benin |
| Bhutan |
| Bolivia (Plurinational State) |
| Bosnia and Herzegovina |
| Botswana |
| Brazil |
| Burkina Faso |
| Burundi |
| Cabo Verde |
| Cambodia |
| Cameroon |
| Central African Republic |
| Chad |
| Chile |
| China |
| China, Taiwan Province of |
| Colombia |
| Comoros |
| Congo |
| Costa Rica |
| CÃ´te d'Ivoire |
| Croatia |
| Cuba |
| Cyprus |
| Democratic Republic of the Congo |
| Dominica |
| Dominican Republic |
| Ecuador |
| Egypt |
| El Salvador |
| Eritrea |
| Eswatini |
| Ethiopia |
| Fiji |
| Gabon |
| Gambia |
| Ghana |
| Guadeloupe |
| Guatemala |
| Guinea |
| Guinea-Bissau |
| Guyana |
| Haiti |
| Honduras |
| India |
| Indonesia |
| Iran (Islamic Republic) |
| Iraq |
| Jamaica |
| Jordan |
| Kazakhstan |
| Kenya |
| Kiribati |
| Kyrgyzstan |
| Lao People's Democratic Republic |
| Lebanon |
| Lesotho |
| Liberia |
| Libya |
| Madagascar |
| Malawi |
| Malaysia |
| Mali |
| Mauritania |
| Mexico |
| Mongolia |
| Montenegro |
| Morocco |
| Mozambique |
| Myanmar |
| Namibia |
| Nicaragua |
| Niger |
| Nigeria |
| Palestine |
| Oman |
| Pakistan |
| Panama |
| Papua New Guinea |
| Paraguay |
| Peru |
| Philippines |
| Republic of Moldova |
| Rwanda |
| Saint Vincent and the Grenadines |
| Samoa |
| Sao Tome and Principe |
| Senegal |
| Serbia |
| Sierra Leone |
| Solomon Islands |
| Somalia |
| South Africa |
| South Sudan |
| Sri Lanka |
| Sudan |
| Suriname |
| Syrian Arab Republic |
| Tajikistan |
| Thailand |
| Timor-Leste |
| Togo |
| Tonga |
| Trinidad and Tobago |
| Tunisia |
| Turkey |
| Turkmenistan |
| Uganda |
| Ukraine |
| United Republic of Tanzania |
| Uruguay |
| Uzbekistan |
| Vanuatu |
| Venezuela (Bolivarian Republic) |
| Viet Nam |
| Western Sahara |
| Yemen |
| Zambia |
| Zimbabwe |
