# Supplementary material for: Global crop waste burning – micro-biochar; how a small community development organization learned experientially to address a huge problem one tiny field at a time
Source: Sustain Earth. 2020 Nov 23;3(1):18. doi: 10.1186/s42055-020-00037-y (PMC7680978; doi:10.1186/s42055-020-00037-y)
Supplement: Supplementary file 2 — Additional file 2. [file 42055_2020_37_MOESM2_ESM.docx]

**Attachment 2: Crop production to waste ratios**

Some crops are “clean” producing little waste relative to the food value they generate; others are dirty. I have used the following ratios to convert FAO crop production figures into crop waste amounts:

Barley, etc. 1 tonne of cereal/coarse grain/grain “NES” (not elsewhere specified) produces 1 tonne of waste (Exception noted below, corn, millet, rice, wheat,) (Coarse grains include barley, buckwheat, canary seed, fonio, millet, rye, oats, sorghum, and triticale)

Cassava 1 tonne of cassava produces 100 kg of cassava peal waste

Coconut 1 tonne of coconut produces 650 kg of husk and shell waste

Corn 1 tonne of corn produces 4 tonnes of stalk, cob and husk field waste

Vegetables 1 tonne of fresh veggies produces 1 tonne of field waste

Ground nut 1 tonne of ground nuts produces 3.5 tonnes of hay and other field waste

Millet 1 tonne of millet produces 2 tonnes of stalk and other field waste

Plantains 1 tonne of plantains (or bananas) produces 2 tonnes of waste

Potatoes 1 tonne of potatoes/roots/tubers produces 200 kg waste (not including field losses)

Rice 1 tonne of rice produces 1.22 tonnes of straw and husk waste

Soy 1 tonne of soy beans produces 1.8 tonnes of straw and shell field waste

Sugar beet 1 tonne of sugar beet produces 200-300 kg of field waste

Sugar cane I tonne of sugar cane produces 200 kg of waste (leaf only)

Tomatoes 1 tonne of tomatoes produces 200 kg of waste

Wheat 1 tonne of wheat produces 1.17 tonnes of waste
