# Supplementary material for: Global crop waste burning – micro-biochar; how a small community development organization learned experientially to address a huge problem one tiny field at a time
Source: Sustain Earth. 2020 Nov 23;3(1):18. doi: 10.1186/s42055-020-00037-y (PMC7680978; doi:10.1186/s42055-020-00037-y)
Supplement: Supplementary file 3 — Additional file 3. [file 42055_2020_37_MOESM3_ESM.docx]

**Attachment 3: Crop waste, emissions from crop waste burning, and impacts**

According to FAOSTAT, total crop production by the included countries runs about 10 billion tonnes annually. Because many crops generate more waste than edible produce, waste production is greater than 10 billion tonnes, but not astronomically so. Combined cereals and coarse grains totaled 2 billion tonnes in 2017; combined waste from cereals and coarse grains totaled 4.2 billion tonnes. Applying this ratio to all crops, total 2017 crop field waste was 21 billion tonnes.

Estimates of how much crop field waste developing world farmers burn still range from 50 to 90 percent. The higher estimate may apply in some places, justified by the often-immense brush fires touched off by open field burning. Still, the best, hard data based study I know (conducted in Mae Chaem District, North Thailand by researchers from Mahidol University in 2018) [8] concluded that only 41 percent of corn stalk was burned. Add this to the large percentage of corncob and husk burned, and the total corn field waste burn probably ran 50-55 percent. Because in many places corn stover (stalk, husk, cob), for example, is used for cooking, construction, or feeding animals, the lower, 50 percent figure is used here.

If developing world farmers burn 50 percent of crop waste – 10.5 billion tonnes – what are the consequences for the climate, environment and public health?

We worry most about four emissions - CO, CO_2_, CH_4_ (methane), NOx, and PM2.5, which can be classified by concern: Climate, Environment and Public Health. For emission factors (EFs), I use S. Akagi, et al., “Emission factors for open and domestic biomass burning for use in atmospheric models,” Atmospheric Chemistry and Physics, 2011: 4039-4071. (There are many alternative EFs available for, for example, rice straw or corn stalk at specific locations. I have not found, however, another source that systematically combines EFs for all emissions for all open field burning everywhere. Such a source is required here, because this exercise is meant to apply to open field burning of all crop residues. Likewise, I have chosen to use the US EPA Global Warming Potentials (GWP). These are 100-year measures; nonetheless, they appear to be the most commonly used in the literature.

| **Emission** | **EF*** | **GWP** |
| --- | --- | --- |
| CO_2_ | 1,585 | 1 |
| CO | 102 |  |
| CH_4_ | 5.82 | 25 |
| NH_3_ | 2.17 |  |
| NOx | 3.11 | 298 |
| PM_2.5_ | 6.26 |  |

*Measured in g/kg

**Climate change**

- **CO_2_** is the most talked about and most emitted climate change gas. CO_2_, however, is also the most problematic of the byproducts of open field burning. To grow, crops must remove CO_2_ from the atmosphere, saving the carbon and releasing the oxygen. Burning 10.5 billion tonnes of crop waste releases **16.6 billion tonnes of CO_2_** into the air. (Of which, 12.5 billion tonnes is oxygen and 4.5 billion tonnes carbon, some of which is carbon neutral.)
- **CO_2_e** (carbon dioxide equivalent) I have conservatively reduced to methane (CH_4_) and NOx, both largely the product of the low temperature of smoldering field fires. Burning 10.5 billion tonnes of crop waste emits ((10,500,000,000 x 5.82)/1000) = 61,110,000 tonnes of methane and ((10,500,000,000 x 3.11)/1,000) = 32,655,000 tonnes of NOx. **CO_2_e** = (61,110,000 x 25) + (32,655,000 x 298) = (1,527,750,000) + (9,731,190,000) = **11,258,940,000 tonnes of CO_2_e**.

**Environment**

- **Smog** forms in the lower atmosphere because of reactions caused when “smog precursor” gases absorb energy from sunlight. WHO estimates that smog kills more than 3 million people per year, although it does not attribute most smog to crop fires. In Beijing, however, as much as 50% of the toxic mix over the city comes from crop fires in June/July, the burning season for rice straw. The three most important smog precursors are CO, NH_3_ (ammonia) and NOx. Primary smog precursors emitted are CO = (10,500,000,000 x 102)/1,000) = 1,071,000,000 tonnes, NH_3_ = (10,500,000,000 x 2.17)/1,000 = 22,785,000 tonnes, and NOx = (10,500,000,000 x 3.11)/1,000) = 32,655,000 tonnes or a combined **1,126,440,000 tonnes of smog precursors.**

**Public health**

- WHO classifies **PM2.5** (particulate matter 2.5 microns or smaller in diameter) as the fifth most important killer in the world today. It kills more people annually than dengue, hepatitis B, HIV, malaria and TB *combined*. PM2.5 is so small that it passes through the walls of the lung into the blood stream and settles in the brain, heart, liver and other organs. PM2.5 not only causes liver and lung cancers, but cirrhosis, COPD, heart attacks and strokes. Burning 10.5 billion tonnes of crop waste generates (10,500,000,000 x 6.26)/1,000 = 65,730,000‬ tonnes of PM2.5. (If you have a hard time imaging the weight of smoke, you are not alone. A simple trick is to remember that 1 kg of smoke is equivalent to the smoke of 71,429 cigarettes, although most of the particulates from cigarette smoke are larger than PM2.5 and remain lodged in the lungs.)
